# DO NOT BECOME A BURDEN: ACTIVATION AND DISENGAGEMENT PRESCRIPTIVE AGE STEREOTYPES

**DOI:** 10.1093/geroni/igz038.2751

**Published:** 2019-11-08

**Authors:** Clara de Paula Couto, Klaus Rothermund

**Affiliations:** Friedrich-Schiller University Jena, Jena, Thuringen, Germany

## Abstract

Prescriptive age stereotypes encompass activation (active-aging) and disengagement expectations (succession-consumption-identity). We investigated whether activation and disengagement represent opposite stereotypes or whether they exemplify the overarching norm that older adults should not become a burden to other people and society. Based on data of the Ageing-as-Future project (N=743 German participants, 40-90 years old) our findings support the idea that activation and disengagement represent a single superordinate prescriptive age stereotype: (a) items assessing prescriptive age stereotypes form a single factor comprising activation and disengagement, (b) activation and disengagement show an increase in the strength of personal endorsement over the lifespan, demonstrating an internalization of these stereotypes as people become older, and (c) relations to reference variables show that internalized prescriptive stereotypes are more strongly associated with preparation for age-related changes, reflecting an internalization of the norm that one should take individual responsibility for their age rather than enjoying life in old age.

